# Drug Susceptibility Testing of 31 Antimicrobial Agents on Rapidly Growing Mycobacteria Isolates from China

**DOI:** 10.1155/2015/419392

**Published:** 2015-08-13

**Authors:** Hui Pang, Guilian Li, Xiuqin Zhao, Haican Liu, Kanglin Wan, Ping Yu

**Affiliations:** ^1^Department of Immunology, Xiangya School of Medicine, Central South University, Changsha, Hunan 410078, China; ^2^Department of Immunology, Changzhi Medical College, Changzhi, Shanxi 046000, China; ^3^State Key Laboratory for Infectious Disease Prevention and Control, National Institute for Communicable Disease Control and Prevention, Chinese Center for Disease Control and Prevention, Beijing 102206, China; ^4^Collaborative Innovation Center for Diagnosis and Treatment of Infectious Diseases, Hangzhou, Zhejiang 310000, China

## Abstract

*Objectives*. Several species of rapidly growing mycobacteria (RGM) are now recognized as human pathogens. However, limited data on effective drug treatments against these organisms exists. Here, we describe the species distribution and drug susceptibility profiles of RGM clinical isolates collected from four southern Chinese provinces from January 2005 to December 2012. *Methods*. Clinical isolates (73) were subjected to *in vitro* testing with 31 antimicrobial agents using the cation-adjusted Mueller-Hinton broth microdilution method. The isolates included 55 *M. abscessus*, 11 *M. fortuitum*, 3 *M. chelonae*, 2 *M. neoaurum*, and 2 *M. septicum* isolates. *Results*. *M. abscessus* (75.34%) and *M. fortuitum* (15.07%), the most common species, exhibited greater antibiotic resistance than the other three species. The isolates had low resistance to amikacin, linezolid, and tigecycline, and high resistance to first-line antituberculous agents, amoxicillin-clavulanic acid, rifapentine, dapsone, thioacetazone, and pasiniazid. *M. abscessus* and *M. fortuitum* were highly resistant to ofloxacin and rifabutin, respectively. The isolates showed moderate resistance to the other antimicrobial agents. *Conclusions*. Our results suggest that tigecycline, linezolid, clofazimine, and cefmetazole are appropriate choices for *M. abscessus* infections. Capreomycin, sulfamethoxazole, tigecycline, clofazimine, and cefmetazole are potentially good choices for *M. fortuitum* infections. Our drug susceptibility data should be useful to clinicians.

## 1. Introduction

Nontuberculous mycobacteria (NTM) form a large class within the Mycobacteriaceae family. More than 100 NTM species are found in soil, potable water, food, and animals [1]. In China, the proportion of NTM among all mycobacterial isolates has increased from 11.1% to 22.9% according to National surveys conducted in 1990 and 2010 [2]. Thus, the rising percentage of NTM in China is now an important public health concern [1, 2].

NTM can be classified into rapidly growing mycobacteria (RGM) and slowly growing mycobacteria (SGM). More than 50 RGM species are able to produce mature colonies on agar plates within 7 days [3]. Many of these are important human pathogens that cause pulmonary and soft tissue infections and various other infections [4, 5]. RGM comprise a diverse group of species, including* M. abscessus*,* M. fortuitum*,* M. chelonae*, and various rare species. Most studies have shown that* M. abscessus* accounts for 80% of the lung disease caused by RGM, and after* M. fortuitum*,* M abscessus* is the second most common RGM to cause extrapulmonary disease [3, 6].

Diagnosing and treating RGM diseases is challenging for clinicians [6, 7]. Over the past few decades, RGM infections have been diagnosed based on a patient's clinical characteristics, risk factors, and the results of antimicrobial susceptibility testing [3]. However, drug susceptibility patterns vary greatly between RGM species and optimally therapeutic regimens have not been established [3, 6].

In this study, the antimicrobial susceptibility of 73 clinical RGM isolates and their corresponding standard strains were tested with 31 antibiotics. The tests were based on the recommendations of the Clinical and Laboratory Standards Institute (CLSI) [8] for determining the clinical criteria for therapeutic treatment of RGM infections.

## 2. Materials and Methods

### 2.1. Clinical Isolates and Reference Strains

During the period from January 2005 to December 2012, RGM clinical strains were isolated from the sputum specimens of suspected tuberculosis patients in Fujian, Hunan, Gansu, and Sichuan Provinces of China. Eighteen clinical strains were isolated in 2005, 6 in 2006, 8 in 2009, 25 in 2010, 8 in 2011, and 8 in 2012. International-standard RGM species were used as the corresponding quality control strains for the clinical isolates tested herein (i.e.,* M. abscessus *ATCC19977,* M. chelonae* ATCC35752,* M. fortuitum* DSM44220,* M. neoaurum* ATCC25795, and* M. septicum* DSM44393).

### 2.2. Species Identification

Species identification of the isolates was conducted by sequence analysis of the* hsp65* gene. When an* hsp65* sequence match was less than 97%, the* rpoB* gene and the 16S–23S internal transcribed spacer region were also sequenced [9, 10]. PCR products were sequenced by the Beijing Tsingke Bio Tech Co. Ltd. (Beijing, China). The sequences obtained were compared with those in the GenBank (National Center for Biotechnology Information: http://www.ncbi.nlm.nih.gov/) DNA sequence database; species identification was confirmed if a 97% match was achieved [9, 10].

### 2.3. RGM Growth Medium

The strains tested were cultured using Difco Middlebrook 7H10 Agar (BD company). The medium was prepared as follows. First, 19 g of 7H10 (powder) was suspended in 900 mL of purified water containing 5 mL of glycerol and then mixed thoroughly. Next, the powder was completely dissolved by heating with frequent agitation for 1 min and then sterilized at 121°C for 10 min. Last, 100 mL of Middlebrook OADC enrichment solution (BD, Franklin Lakes, NJ, USA) was added aseptically to the medium after cooling to 50–55°C.

### 2.4. Medium for Drug Susceptibility Testing of RGM

The medium used for antimicrobial susceptibility testing of RGM was BBL cation-adjusted Mueller-Hinton (CAMH) Broth (BD, Franklin Lakes, NJ, USA). The medium was prepared by suspending 22 g of powder in 1 L of purified water and autoclaving the bottle at 121°C for 10 min, followed by supplementation with 20–25 mg/L of calcium and 10–12.5 mg/L of magnesium.

### 2.5. Drug Susceptibility Tests

The strains were grown on 7H10 agar and incubated at 37°C in ambient air. Drug susceptibility tests were performed using the broth microdilution method according to CLSI recommendations [6]. Tests on the strains were repeated at least twice using 96-well microplates. The final minimum inhibitory concentration (MIC) of each drug used for each strain was the average value of the two tests. Bacterial inocula were adjusted with normal saline to a density of a 0.5 McFarland standard with an organism density of approximately 1 × 10^7^ colony forming units (CFU)/mL. Fifty microliters of the suspension, added to 10 mL of CAMH broth, was vortexed thoroughly to make a 1 : 200 bacterial dilution. First, 100 *μ*L of CAMH medium was added to each well of a 96-well microplate, except for the first well of the each row. CAMH medium (180 *μ*L) was added to the first well of every row, followed by a 20 *μ*L aliquot of a drug solution. The thoroughly mixed solution in the first well was serially diluted into the next well, and so on up to the 11th well. The 12th well in every row was a blank control. Second, 100 *μ*L of the bacterial dilution was added to the wells of the 96-well microplate. The final volume in each row was 200 *μ*L. Finally, the 96-well microplate was sealed in a plastic bag and incubated at 37°C. The concentration ranges for rifampicin, isoniazid, ethambutol, streptomycin, tobramycin, sulfamethoxazole, dapsone, amoxicillin-clavulanic acid, cefoxitin, cefmetazole, thioacetazone, pasiniazid, minocycline, doxycycline, tigecycline, and meropenem were all 0.25–256 *μ*g/mL, those of amikacin, kanamycin, capreomycin, ofloxacin, ciprofloxacin, levofloxacin, moxifloxacin, sparfloxacin, clarithromycin, azithromycin, roxithromycin, clofazimine, rifapentine, and rifabutin were 0.03–32 *μ*g/mL, and the concentration of linezolid was 0.06–64 *μ*g/mL. All the drugs were purchased from Sigma-Aldrich (St. Louis, MO). The following two negative controls were used: CAMH broth plus inoculum (drug-free control), which was used to decide the optimal time to add Alamar blue to the assay; the other was only CAMH broth, which was used to decide the interference level of CAMH to Alamar blue. The plates were checked after 72 h. If the drug-free growth control showed sufficient bacterial growth, the indicator (20 *μ*L of Alamar Blue and 50 *μ*L of sterile 5% Tween-80) turned pink. Generally, the minimal inhibitory concentration (MIC) value was read on day 3 or 4 after addition of the inoculum. If bacterial growth in the drug-free control was insufficient on day 5, the test was repeated. The MIC values for clarithromycin were evaluated 3 to 5 days after inoculation and were incubated for a further 14 days at 37°C for the final reading.

MIC values were defined as the lowest concentration of drug that inhibited the visible growth of the isolates tested. MIC_50_ and MIC_90_ values were defined as the drug concentrations at which 50% and 90% of the isolates tested showed no visible growth, respectively. The MIC breakpoints of antibiotics displaying susceptibility, intermediate susceptibility, and resistance were interpreted by the World Health Organization (WHO) [11] and CLSI guidelines [8], except for sparfloxacin [12], clofazimine [13], azithromycin [14], roxithromycin [14], amoxicillin-clavulanic acid [15], cefmetazole [16], rifapentine [17], rifabutin [17], dapsone [18], and thioacetazone [19] (Table 1).

### 2.6. Statistical Analysis

The data were analyzed by SPSS17.0 software. The drug susceptibility percentages and the MIC_50_ and MIC_90_ among the antimicrobial agents tested were determined for the five species of RGM isolates (*M. abscessus*,* M. chelonae*,* M. fortuitum*,* M. neoaurum*, and* M. septicum*).

## 3. Results

Among the 73 clinical isolates, 55 (75.34%) were* M. abscessus*, 11 (15.07%) were* M. fortuitum*, 3 (4.11%) were* M. chelonae*, 2 (2.74%) were* M. neoaurum*, and 2 (2.74%) were* M. septicum*. Of the isolates, 63 were from Fujian, 7 were from Hunan, 2 were from Gansu, and 1 was from Sichuan.

The antimicrobial susceptibility profiles of the five reference RGM strains are shown in Table 2. The strains were highly resistant to the four first-line antituberculous agents tested on them, especially* M. chelonae*,* M. abscessus,* and* M. fortuitum*. We found that aminoglycoside antibiotics including amikacin, kanamycin, capreomycin, and tobramycin, were effective antimicrobials for the RGM species. However,* M. abscessus* was resistant to tobramycin and* M. chelonae* was resistant to kanamycin and capreomycin. Fluoroquinolones (including ofloxacin, ciprofloxacin, levofloxacin, sparfloxacin, and moxifloxacin) also exhibited favorable* in vitro* activities against the standard RGM strains. However,* M. chelonae* was resistant to all four of the fluoroquinolones we tested.* M. chelonae* and* M. abscessus* were not susceptible to minocycline and doxycycline, but were susceptible to tigecycline. Clarithromycin, azithromycin, and roxithromycin all exhibited favorable* in vitro* activities (except for azithromycin) against* M. septicum*. The reference species were not susceptible to amoxicillin-clavulanic acid but were susceptible or moderately susceptible to cefoxitin and cefmetazole, apart from* M. chelonae* and* M. septicum*, which were not susceptible to cefoxitin. Unlike* M. chelonae*, the RGM species were susceptible to meropenem. Rifapentine had a more favorable MIC than rifabutin against* M. chelonae*,* M. abscessus,* and* M. fortuitum*. Linezolid, clofazimine, and sulfamethoxazole were highly active against the standard RGM organisms. However, dapsone, thioacetazone, and pasiniazid displayed poor activities against the reference species.

The percentage of* in vitro* drug susceptibility values of the 31 antibacterial agents against the 73 clinical RGM isolates is shown in Table 3. Among the four first-line antituberculous drugs, no (0/73) strains were susceptible to isoniazid and 3 (4.11%), 2 (2.74%), and 4 (5.48%) strains were susceptible to rifampicin, ethambutol, and streptomycin, respectively. However, the* M. chelonae* isolates were less resistant to rifampicin than* M. abscessus* and* M. fortuitum. *Aminoglycosides and fluoroquinolones displayed a range of activities against the RGM isolates. Amikacin displayed the highest activity (72/73, 98.63%), while moxifloxacin displayed a range of activities (57/73, 78.08%). Tigecycline (70/73, 95.89%) had much higher activity against the isolates than minocycline (30/73, 41.10%) and doxycycline (25/73, 34.25%). Clarithromycin (48/73, 65.75%), azithromycin (53/73, 72.60%), and roxithromycin (48/73, 65.75%) showed various* in vitro* activities against the RGM isolates. Meropenem (52/73, 71.23%) exhibited good activity against the strains tested. Cefmetazole (62/73, 84.93%) was more active than cefoxitin (49/73, 67.12%) and amoxicillin-clavulanic acid (11/73, 15.07%). In contrast, linezolid (71/73, 97.26%) was highly active against the majority of the RGM isolates. Clofazimine (47/73, 64.38%) and sulfamethoxazole (56/73, 76.71%) inhibited the majority of isolates. Rifapentine (5/73, 6.85%) and rifabutin (26/73, 35.62%), which are rifamycin derivatives, were better than rifampicin. However, dapsone (5/73, 6.85%), thioacetazone (0/73, 0%), and pasiniazid (0/73, 0%) displayed poor activities against the RGM isolates.

The MICs, MIC_50_, and MIC_90_ ranges for each antimicrobial agent tested against each RGM species are shown in Table 4. Based on the MIC_90_ values of the isolates, capreomycin was 8 *μ*g/mL for* M. abscessus* and 2 *μ*g/mL for* M. fortuitum*. The MIC_50_ value for levofloxacin was 8 *μ*g/mL for* M. abscessus* and 2 *μ*g/mL for* M. fortuitum*. Clofazimine had very high activity, with MIC_90_ values of 8 *μ*g/mL for* M. abscessus* and 2 *μ*g/mL for* M. fortuitum*. The MIC_50_ and MIC_90_ values of doxycycline were 16 *μ*g/mL and 32 *μ*g/mL, respectively, for* M. abscessus*, and 64 *μ*g/mL and 256 *μ*g/mL, respectively, for* M. fortuitum*. Azithromycin had better activity against* M. abscessus* than* M. fortuitum*, with MIC_50_ values of 0.5 *μ*g/mL and 2 *μ*g/mL, respectively.

## 4. Discussion

With the development of improved microbiological and laboratory techniques, more RGM have been identified [20]. Effective treatment of RGM-related diseases is challenging to physicians because it is not obvious which drugs should be selected. In this study, the susceptibilities of 73 clinical RGM isolates and their corresponding reference RGM strains were examined for 31 antimicrobial agents using CAMH broth microdilution methodology.

Some studies have shown that* M. abscessus*,* M. fortuitum,* and* M. chelonae *are important human pathogens among RGM isolates [21–24]. Here, we showed that* M. abscessus *(75.34%) is the predominant RGM species in the 73 clinical isolates, followed by* M. fortuitum *(15.07%). Both organisms were susceptible to amikacin, linezolid, tigecycline, cefmetazole, capreomycin, moxifloxacin, macrolides, and carbapenems, but were highly resistant to the first-line antituberculous drugs, dapsone, thioacetazone, and pasiniazid. The percentage of resistance to numerous drugs was higher in* M. abscessus* than in* M. fortuitum*, except for moxifloxacin, minocycline, doxycycline, roxithromycin, cefmetazole, and rifabutin. In a recent report, amikacin and clarithromycin were the optimal choices against infection with* M. abscessus* [25]. Additionally, quinolones and trimethoprim-sulfamethoxazole were effective against* M. fortuitum* [25]. In the present study, Amikacin was the most active drug against* M. abscessus*. Furthermore, we found that amikacin, capreomycin, and linezolid had the highest antibacterial activities against* M. fortuitum*.

Previous studies have reported that numerous RGM strains were highly resistant to the first-line antituberculous agents [26, 27]. Our data confirms this finding. Elsewhere, researchers have shown that dapsone had little activity against RGM isolates [18]. Thioacetazone is used mainly as an antituberculous agent but has variable activity, and the drug was formerly used in conjunction with isoniazid [19]. RGM strains have been shown to be highly resistant to pasiniazid [28]. Our data shows that dapsone had little activity against* M. abscessus* and* M. fortuitum* isolates, while thioacetazone and pasiniazid had no activity against any of the RGM organisms.

Aminoglycosides and quinolones, which are second-line antituberculous drugs, have good activities against RGM strains [28–30]. In our study, amikacin was found to have potential to be effective for treatment of RGM diseases and showed higher activity than the other aminoglycoside antibiotics we tested. However, a higher percentage of* M. chelonae* isolates were sensitive to tobramycin than* M. abscessus*, although the sample size of the latter was smaller. The third generation fluoroquinolone drugs levofloxacin and sparfloxacin displayed higher activities than ofloxacin. Moxifloxacin, a fourth generation fluoroquinolone, displayed higher activity than the third generation ones [30]. Quinolones exhibited better activity against* M. fortuitum* than* M. abscessus*, especially levofloxacin, against which* M. fortuitum* was more susceptible than* M. abscessus*.

Minocycline, doxycycline, and tigecycline represent the newest tetracycline derivatives [31]. A recent study [26] showed that NTM displayed ~50% susceptibility to doxycycline and minocycline, but in our research susceptibility to these two drugs was more than 20% (Table 3). This finding may reflect the small sample number of* M. fortuitum* in this study. Tigecycline displayed activity against RGM organisms [26]. We found that tigecycline had more activity than minocycline and doxycycline, with lower MIC_50_ and MIC_90_ values.

RGM strains have been shown to be susceptible to the newer generation of macrolide antibiotics (i.e., clarithromycin, azithromycin, and roxithromycin) [25, 26, 30]. This drug class is a good alternative for treating RGM species because of its high activity and oral formulations. Clinical experience shows that azithromycin toxicity is dose dependent and most adult patients with* M. avium* complex (MAC) lung disease do not tolerate azithromycin doses greater than 300 mg/day because of frequent of adverse events, including gastrointestinal symptoms (primarily diarrhea) and reversible hearing impairment [26]. In our research, the isolates were less susceptible to clarithromycin than to azithromycin, making the latter more applicable in the future. Some reports suggest that clarithromycin resistance can be induced in* M. abscessus* and* M. fortuitum*, and the resistance is associated with erm (41) and erm (39) genes, respectively [26, 32]. The* M. abscessus* complex is subclassified into three closely related subspecies (*M. abscessus*,* M. massiliense,* and* M. bolletii*) [33]. Historically, the* M. fortuitum* group has included three species:* M. fortuitum*,* M. peregrinum*, and an unnamed third biovariant complex [34]. The different subspecies potentially exhibit different drug susceptibilities. Macrolide resistance in* M. abscessus* and* M. fortuitum* will be the subject of our future research.

Cefoxitin and cefmetazole are second and third generation cephalosporin antibiotics and in previous studies cefoxitin was frequently found to have good mycobacterial activity [25, 26, 28, 30]. The isolates were more susceptible to cefmetazole than cefoxitin, so cefmetazole can be used where cefoxitin is ineffective. In addition, the antibacterial mechanism for meropenem, a carbapenem antibiotic, occurs via inhibition of bacterial cell wall synthesis [35]. Carbapenems include imipenem, meropenem, and ertapenem, among others. In previous studies, imipenem was widely used in experiments [25, 26, 30]. Here, among the RGM strains, meropenem was found to have good activity and a lower MIC value than cefoxitin. In clinical work, rifabutin has been used mainly to target SGM, and toxicity to this drug was dose related. Clarithromycin has been shown to increase rifabutin serum levels and this effect was likely to be related to the hepatic metabolism of rifabutin [26]. Our data indicates that rifabutin and rifapentine can be used to treat rifampicin-resistant strains; the RGM isolates have better susceptibility to rifabutin than rifapentine, but rifapentine was more active against* M. chelonae*,* M. abscessus,* and* M. fortuitum*. Otherwise, the rifabutin dose should be reduced when used in combination with clarithromycin to treat infections with RGM strains.

Several studies have shown that linezolid and clofazimine have potent activities against NTM [25, 26, 29, 30, 36]. In our experiments, linezolid had >95% activity against the strains tested and clofazimine > 60% susceptibility for the RGM isolates (with the exception of* M. chelonae*). The small* M. chelonae* sample size probably affected this result. In the future, a larger sample size should be used to determine the MICs of the 31 drugs used in this study.

The RGM isolates used in our research were from four different Chinese provinces. Most of them (86.30%) were from Fujian Province, making it important to obtain more samples from different geographical areas in the future.

The data presented here suggest that tigecycline or linezolid combined with clofazimine or cefmetazole should be the most efficacious drug combination for treating* M. abscessus* infections. For* M. fortuitum,* capreomycin, sulfamethoxazole, tigecycline, clofazimine, and cefmetazole in combination may be a good choice.

## 5. Conclusions

In summary, our data provide useful information on antibiotics that are effective against RGM and this information may help to identify suitable therapy for patients infected with such organisms. Future studies should address whether combining two or more antimicrobial agents for treatment of RGM infections is better than treatment with a single drug alone.

## Figures and Tables

**Table 1:** MIC (*μ*g/mL) breakpoints of 31 antimicrobial agents.

Antibacterial agents	MIC breakpoints		
	Susceptibility	Intermediate susceptibility	Resistance
Rifampicin	—	—	⩾1 [8]
Isoniazid	—	—	⩾1 [8]
Ethambutol	—	—	⩾4 [8]
Streptomycin	—	—	⩾5 [8]
Amikacin	⩽16	32	⩾64 [8]
Kanamycin	—	—	⩾4 [11]
Capreomycin	—	—	⩾2.5 [11]
Tobramycin	⩽2	4	⩾8 [8]
Ofloxacin	—	—	⩾2 [11]
Ciprofloxacin	⩽1	2	⩾4 [8]
Levofloxacin	⩽2	4	⩾8 [8]
Sparfloxacin	⩽1	2	⩾4 [12]
Moxifloxacin	⩽1	2	⩾4 [8]
Linezolid	⩽8	16	⩾32 [8]
Clofazimine	—	—	⩾1 [13]
Sulfamethoxazole	⩽38	—	⩾76 [8]
Minocycline	⩽1	2–4	⩾8 [8]
Doxycycline	⩽1	2–4	⩾8 [8]
Tigecycline	⩽1	2–4	⩾8 [8]
Clarithromycin	⩽2	4	⩾8 [8]
Azithromycin	⩽2	4	⩾8 [14]
Roxithromycin	⩽2	4	⩾8 [14]
Amoxicillin-clavulanic acid	⩽8/4	16/8	⩾32/16 [15]
Cefoxitin	⩽16	32–64	⩾128 [8]
Cefmetazole	⩽16	32	⩾64 [16]
Meropenem	⩽4	8–16	⩾32 [8]
Rifapentine	—	—	⩾1 [17]
Rifabutin	—	—	⩾1 [17]
Dapsone	—	—	⩾4 [18]
Thioacetazone	—	—	⩾8 [19]
Pasiniazid	—	—	⩾2 [11]

**Table 2:** MIC (*μ*g/mL) results of the antimicrobial susceptibility tests for five rapidly growing reference mycobacteria.

Sp.	RFP	INH	EMB	SM	AM	KN	CPM	TOB	OF	CIP	LEV	SPA	MXF	LNZ	CLO	SMZ
*M. abscessus*	64	>256	32	16	***1***	***2***	***0.5***	8	4	**2**	**1**	**2**	***1***	***4***	***0.06***	***32***
*M. chelonae*	>256	>256	128	32	***4***	16	16	***2***	32	4	16	16	4	***8***	***0.25***	64
*M. fortuitum*	128	64	256	32	***0.25***	4	***<0.03***	16	***0.13***	***0.03***	***0.03***	***<0.03***	***<0.03***	***8***	***0.03***	***2***
*M. septicum*	16	256	4	16	***0.13***	***0.5***	***<0.03***	***1***	***0.25***	***0.06***	***0.13***	***0.03***	***0.06***	***1***	***0.13***	***1***
*M. neoaurum*	1	>256	8	***0.5***	***0.5***	***0.06***	***<0.03***	***0.25***	***0.06***	***0.06***	***0.03***	***<0.03***	***<0.03***	***0.5***	***0.03***	***0.5***

Sp.	MIN	DOX	TIG	CLR	AZM	ROX	AMC	FOX	CMZ	MEM	RFT	RFB	DAP	THI	PASI	

*M. abscessus*	8	32	**4**	***<0.03***	***<0.03***	***0.03***	>256	**32**	**32**	64	***0.13***	32	64	16	32	
*M. chelonae*	64	>32	***1***	***0.06***	***0.5***	***2***	>256	128	***<0.25***	>256	***0.13***	32	32	>256	>256	
*M. fortuitum*	8	***0.03***	***0.5***	***1***	***2***	**4**	>256	**32**	***4***	***4***	1	16	8	256	2	
*M. septicum*	32	8	***1***	***1***	16	**4**	>256	128	***1***	**16**	***0.13***	***<0.03***	32	256	4	
*M. neoaurum*	***0.03***	***0.06***	***0.03***	***1***	***1***	***2***	32	***8***	***1***	***0.5***	***0.06***	***0.13***	4	>256	64	

Note 1: *M. abscessus*: ATCC19977;* M. chelonae*: ATCC35752; *M. fortuitum*: DSM44220; *M. neoaurum*: ATCC25795; and *M. septicum*: DSM44393.

Note 2: INH: isoniazid; RFP: rifampicin; EMB: ethambutol; SM: streptomycin; AM: amikacin; KN: kanamycin; CPM: capreomycin; TOB: tobramycin; OF: ofloxacin; CIP: ciprofloxacin; LEV: levofloxacin; SPA: sparfloxacin; MXF: moxifloxacin; LNZ: linezolid; CLO: clofazimine; SMZ: sulfamethoxazole; CLR: clarithromycin; AZM: azithromycin; ROX: roxithromycin; MIN: minocycline; DOX: doxycycline; TIG: tigecycline; AMC: Amoxicillin-clavulanic Acid; FOX: cefoxitin; CMZ: cefmetazole; MEM: meropenem; RPT: rifapentine; RBT: rifabutin; DAP: dapsone; THI: thioacetazone; PASI: pasiniazid.

Note 3: Bold, italic values indicate drug susceptibility. Values shown in bold indicate moderate drug susceptibility.

**Table 3:** * In vitro* drug susceptibility percentage per species for rapidly growing mycobacteria isolates.

Drugs	Species					Total (*n* = 73) (%)
	*M. abscessus* (*n* = 55) (%)	*M. fortuitum* (*n* = 11) (%)	*M. chelonae* (*n* = 3) (%)	*M. septicum* (*n* = 2) (%)	*M. neoaurum* (*n* = 2) (%)	
INH	0 (0)	0 (0)	0 (0)	0 (0)	0 (0)	0 (0)
RFP	3 (5.46)	0 (0)	0 (0)	0 (0)	0 (0)	3 (4.11)
EMB	2 (3.64)	0 (0)	0 (0)	0 (0)	0 (0)	2 (2.74)
SM	3 (5.46)	0 (0)	0 (0)	0 (0)	1 (50.00)	4 (5.48)
AM	55 (100.00)	11 (100.00)	3 (100.00)	2 (100.00)	1 (50.00)	72 (98.63)
KN	27 (49.09)	5 (45.45)	1 (33.33)	1 (50.00)	0 (0)	34 (46.58)
CPM	41 (74.55)	11 (100.00)	1 (33.33)	2 (100.00)	0 (0)	55 (75.34)
TOB	25 (45.45)	6 (54.55)	3 (100.00)	1 (50.00)	0 (0)	35 (47.94)
OF	9 (16.36)	5 (45.45)	0 (0)	2 (100.00)	2 (100.00)	18 (24.66)
CIP	24 (43.64)	5 (45.45)	1 (33.33)	2 (100.00)	1 (50.00)	33 (45.21)
LEV	26 (47.27)	8 (72.73)	2 (66.67)	2 (100.00)	1 (50.00)	39 (53.42)
SPA	23 (41.82)	6 (54.55)	3 (100.00)	2 (100.00)	1 (50.00)	35 (47.94)
MXF	43 (78.18)	8 (72.73)	3 (100.00)	2 (100.00)	1 (50.00)	57 (78.08)
LNZ	53 (96.36)	11 (100.00)	3 (100.00)	2 (100.00)	2 (100.00)	71 (97.26)
CLO	35 (63.64)	8 (72.73)	1 (33.33)	2 (100.00)	1 (50.00)	47 (64.38)
SMZ	41 (74.55)	10 (90.91)	2 (66.67)	2 (100.00)	1 (50.00)	56 (76.71)
MIN	24 (43.64)	3 (27.27)	2 (66.67)	1 (50.00)	0 (0)	30 (41.10)
DOX	19 (34.55)	3 (27.27)	2 (66.67)	0 (0)	1 (50.00)	25 (34.25)
TIG	53 (96.36)	10 (90.91)	3 (100.00)	2 (100.00)	2 (100.00)	70 (95.89)
CLR	37 (67.27)	7 (63.64)	2 (66.67)	1 (50.00)	1 (50.00)	48 (65.75)
AZM	43 (78.18)	6 (54.55)	2 (66.67)	0 (0)	2 (100.00)	53 (72.60)
ROX	38 (69.09)	7 (63.64)	2 (66.67)	0 (0)	1 (50.00)	48 (65.75)
AMC	10 (18.18)	0 (0)	1 (33.33)	0 (0)	0 (0)	11 (15.07)
FOX	38 (69.09)	9 (81.82)	1 (33.33)	0 (0)	1 (50.00)	49 (67.12)
CMZ	48 (87.27)	9 (81.82)	2 (66.67)	1 (50.00)	2 (100.00)	62 (84.93)
MEM	39 (70.91)	8 (72.73)	1 (33.33)	2 (100.00)	2 (100.00)	52 (71.23)
RPT	4 (7.27)	0 (0)	1 (33.33)	0 (0)	0 (0)	5 (6.85)
RBT	22 (40.00)	1 (9.09)	2 (66.67)	0 (0)	1 (50.00)	26 (35.62)
DAP	4 (7.27)	1 (9.09)	0 (0)	0 (0)	0 (0)	5 (6.85)
THI	0 (0)	0 (0)	0 (0)	0 (0)	0 (0)	0 (0)
PASI	0 (0)	0 (0)	0 (0)	0 (0)	0 (0)	0 (0)

Note: *n*: number of strains tested.

**Table 4:** The MIC range, MIC_50_, and MIC_90_ (*μ*g/mL) per species of rapidly growing mycobacterial isolates for all the antibacterial agents tested.

Drugs	*M. abscessus* (*n* = 55)			*M. fortuitum* (*n* = 11)			Other species (*n* = 7)		
	MIC range	MIC_50_	MIC_90_	MIC range	MIC_50_	MIC_90_	MIC range	MIC_50_	MIC_90_
RFP	0.5–>256	16	128	1–>256	128	>256	2–8	2	8
INH	2–>256	>256	>256	2–>256	>256	>256	16–>256	>256	>256
EMB	1–>256	>256	>256	32–>256	256	>256	128–>256	>256	>256
SM	<0.25–>256	32	256	8–>256	32	128	4–64	32	64
AM	0.06–16	4	8	0.06–16	2	4	0.25–>32	4	8
KN	0.25–>32	4	32	0.5–>32	8	16	2–>32	16	>32
CPM	0.03–16	1	8	0.03–2	0.5	2	0.03–32	4	16
TOB	<0.25–64	8	32	0.5–32	4	32	0.5–32	4	16
OF	0.06–32	8	16	0.06–32	4	16	0.03–8	0.5	8
CIP	0.03–32	4	4	0.06–8	4	8	0.13–8	2	4
LEV	0.03–32	8	8	0.06–8	2	8	0.13–16	4	8
SPA	0.03–32	4	16	0.03–16	2	16	0.06–16	0.25	2
MXF	0.03–16	2	4	0.03–8	1	4	0.06–8	0.5	2
LNZ	0.5–>64	4	16	0.5–16	4	16	0.25–16	4	8
CLO	0.06–>32	0.25	8	0.03–16	0.25	2	0.06–>32	0.5	4
SMZ	0.5–>256	32	>256	4–128	16	64	4–>256	16	256
MIN	<0.25–>256	16	32	1–256	16	64	2–32	16	32
DOX	<0.25–>256	16	32	<0.25–256	64	256	1–64	8	32
TIG	<0.25–8	2	2	<0.25–8	4	4	<0.25–1	0.5	1
CLR	0.03–>32	0.13	>32	0.06–>32	0.25	16	0.03–>32	1	8
AZM	0.03–>32	0.5	>32	0.13–>32	2	>32	0.13–>32	1	32
ROX	0.03–>32	0.25	>32	0.13–32	1	32	0.03–16	8	16
AMC	0.5–>256	>256	>256	256–>256	>256	>256	1–>256	>256	>256
FOX	4–>256	64	256	16–>256	32	128	32–>256	128	256
CMZ	1–128	16	64	8–64	16	64	8–>256	32	64
MEM	<0.25–>256	8	128	<0.25–>256	8	256	0.5–32	4	32
RPT	0.03–>32	8	32	1–>32	8	>32	0.06–32	2	16
RBT	0.03–>32	1	16	0.5–>32	2	16	0.03–4	1	4
DAP	1–>256	16	128	2–>256	64	128	4–256	16	64
THI	8–>256	>256	>256	128–>256	>256	>256	128–>256	>256	>256
PASI	2–>256	256	>256	8–>256	>256	>256	16–>256	>256	>256

Note: *n*: number of strains tested.
